# Circulating Tumor Cells as a Tool to Untangle the Breast Cancer Heterogeneity Issue

**DOI:** 10.3390/biomedicines9091242

**Published:** 2021-09-16

**Authors:** Tania Rossi, Giulia Gallerani, Giovanni Martinelli, Roberta Maltoni, Francesco Fabbri

**Affiliations:** 1 Biosciences Laboratory, IRCCS Istituto Romagnolo per lo Studio dei Tumori (IRST) “Dino Amadori”, 47014 Meldola, Italy; giulia.gallerani@irst.emr.it (G.G.); francesco.fabbri@irst.emr.it (F.F.); 2 Scientific Directorate, IRCCS Istituto Romagnolo per lo Studio dei Tumori (IRST) “Dino Amadori”, 47014 Meldola, Italy; giovanni.martinelli@irst.emr.it; 3 Healthcare Administration, IRCCS Istituto Romagnolo per lo Studio dei Tumori (IRST) “Dino Amadori”, 47014 Meldola, Italy; roberta.maltoni@irst.emr.it

**Keywords:** breast cancer, liquid biopsy, circulating tumor cells, heterogeneity

## Abstract

Breast cancer (BC) is a disease characterized by high degrees of heterogeneity at morphologic, genomic, and genetic levels, even within the same tumor mass or among patients. As a consequence, different subpopulations coexist and less represented clones may have a selective advantage, significantly influencing the outcome of BC patients. Circulating tumor cells (CTCs) represent a rare population of cells with a crucial role in metastatic cascade, and in recent years have represented a fascinating alternative to overcome the heterogeneity issue as a “liquid biopsy”. However, besides the raw enumeration of these cells in advanced epithelial tumors, there are no CTC-based assays applied in the clinical practice to improve personalized medicine. In this review, we report the latest findings in the field of CTCs for intra-tumoral heterogeneity unmasking in BC, supporting the need to deepen their analysis to investigate their role in metastatic process and include the molecular characterization in the clinical practice. In the future, CTCs will be helpful in monitoring patients during treatment, as well as to better address therapeutic strategies.

## 1. Introduction

Breast cancer (BC) is the most diagnosed tumor and the leading cause of tumor-related death among women worldwide [1]. Despite the great progress in BC research, it still remains an undeniable health issue since recurrence can occur even after decades [2], although the precise mechanisms still need to be elucidated.

One of the well-known hallmarks of BC is represented by its high levels of heterogeneity at morphologic [3], genomic [3,4], transcriptomic [5], and proteomic [6] levels, making the choice and monitoring of treatments challenging. This heterogeneity underlies the presence of several subpopulations within the tumor harboring different characteristics, i.e., metastatic potential and treatment resistance [7]. As a result, conventional solid biopsy risks providing information concerning a non-representative sample of the whole tumor, thus excluding the entire spectrum of alterations [8,9].

To overcome this bias, the investigation of circulating markers, among which circulating tumor cells (CTCs), has proven to be a fascinating alternative to dissect this diversity through a liquid biopsy approach [10,11]. The main advantage is that CTCs can be easily recovered from the peripheral blood of patients diagnosed with epithelial tumors, including BC [12], employing minimally invasive techniques. Serial blood sampling allows investigating the evolution of the disease along the course of the patient’s management, monitoring drug response/resistance, and the presence of minimal residual disease [13]. To date, the only FDA-approved CTC-based assay consists of the enumeration of CTCs in peripheral blood of patients diagnosed with metastatic epithelial tumors, including BC. In this setting, the presence of ≥5 CTCs in 7.5 mL of peripheral blood reflects an unfavorable prognosis, but CTCs are extremely rare in early-stage BC and their isolation is challenging [14]. A meta-analysis conducted on 6825 BC patients highlighted that the presence of CTCs had a strong impact on progression and increasing death risk [15]. New progress in CTC molecular analysis, in particular using next-generation sequencing (NGS) approaches both in bulk and at the single-cell level, has contributed to unraveling BC heterogeneity [16,17].

This review summarizes the implications of CTC characterization to overcome BC heterogeneity, emphasizing the relevance of their analysis in tumor surveillance and monitoring. We infer the urgent need to standardize and integrate circulating biomarkers assessment into clinical practice to improve personalized medicine.

## 2. Breast Cancer: A Heterogeneous Disease

One hallmark of BC is its heterogeneity. To date, BC is routinely distinguished in three broad subtypes based on the expression of diagnostic biomarkers: luminal (estrogen (ER) and progesterone (PgR) receptors positive, human epidermal growth factor receptor 2 (HER2) positive or negative), HER2-enriched (ER and PR negative, HER2 positive) and triple-negative (ER, PR, and HER2 negative) BC. Moreover, the World Health Organization (WHO), based on morphologic growth patterns and cytological aspects, identifies at least 20 invasive breast tumor subtypes [3]. More recently, with the advent and improvement of advanced techniques, such as gene expression profiling and NGS, a new taxonomy of BC based on molecular characteristics has been proposed [18]. In recent years, several studies [19,20,21] have tried to uncover through molecular analyses the spectrum of BC alterations, identifying a number of commonly observed point mutations (e.g., *PIK3CA* and *TP53* mutations [22,23]) and copy number gains (e.g., *CCND1* gene [24]). However, due to the wide-range alterations occurring in mammary epithelial cells, BC is considered as a bona fide heterogeneous disease, reason why patients display different disease manifestations and outcome. 

Contextually, in addition to the differences occurring among different patients (inter-tumor heterogeneity), high levels of diversity have been described even within each individual tumor (intra-tumor heterogeneity; ITH) [25], with demonstrated clinical importance. 

Up to now, BC ITH still remains a complex concern since the mechanisms at its basis have both clonal and non-clonal origins [26]. More specifically, the clonal evolution theory assumes changes at the level of oncogenes or tumor-suppressor genes that are transmitted to tumor daughter cells. Such changes include mutations and copy number aberrations (CNAs), as well as epigenetic events such as promoter methylation, hypomethylation of tumor DNA, histone methylation, and deacetylation. Whole-exome techniques proved to give details concerning the presence of subclonal structures and clonal evolution in inflammatory BC, a rare and aggressive subtype characterized by a high mortality rate. This approach led to the identification of subclonal structures sharing mutations on driver genes (*PTEN* and *FBXW7*) [27]. At the same time, cells within the same tumor mass can interact in an autocrine manner with cells from the same or other subclones. Simultaneously, tumor cells are able to interact with cells from the tumor microenvironment (TME) via paracrine communication. Beyond the diversity of tumor cells, the plasticity of TME cells such as tumor-associated macrophages (TAMs), tumor-associated neutrophils (TANs), and myeloid-derived suppressor cells (MDSCs), represent another layer of plasticity further contributing to heterogeneity in BC [28]. A study conducted by Janiszewska et al. demonstrated the cooperation of BC cells from different subclones expressing interleukin-11 (IL11) and vascular endothelial growth factor D (VEGFD) to promote the occurrence of polyclonal metastases with driver and neutral subclones. Strikingly, IL11 orchestrates the interaction with the immune cells of the TME and induces pro-tumorigenic and pro-metastatic neutrophils [29]. Recently, Wang and colleagues explored DNA methylation based on epialleles of one BC patient by sampling different parts of the tumor mass (i.e., tumor core and periphery). They found that the tumor core was characterized by higher levels of epigenetic and transcriptional heterogeneity, and functional analyses revealed that hypoxic TME could have a role in the modulation of the epigenetic status in the tumor. This study has a noteworthy clinical relevance, since methylation markers were also shown to be associated with survival in BC patients [30].

In HER2-enriched BC patients treated with neoadjuvant trastuzumab and chemotherapy, HER2 copy number heterogeneity was shown to impact disease progression and survival, emphasizing the importance of HER2 copy number monitoring to predict the therapy response [31]. Cells exhibiting PIK3CA mutations and HER2 amplification were more frequent in pre-treatment samples of patients who did not benefit from trastuzumab/chemotherapy treatment. In addition, subclones with these alterations were demonstrated to expand in non-responder patients, implying a role of these alterations in therapy resistance and tumor progression [32]. Another highly heterogeneous subtype consists of triple-negative BC (TNBC). TNBC patients displaying higher levels of ITH were reported to be significantly associated with metastasis occurrence and worse metastasis-free survival, suggesting that ITH could serve as a valid prognostic factor [33]. Moreover, resistant clones selected by neoadjuvant chemotherapy (NAC) were shown to be pre-existent before treatment, suggesting an evolutionary model through single-cell analysis [34]. However, when receptor-based classification is used for BC, it is not uncommon to observe receptor conversion (i.e., discordance of receptor expression between primary and metastatic tumor in the same patient), which represents a heavy issue with direct clinical significance making treatment decisions in metastatic BC (mBC) challenging, since addressing therapy exclusively based on the primary tumor characteristics may be misleading. A meta-analysis by Schrjver et al. demonstrates that although percentages among the studies are variable, receptor conversion is a frequent event, and reflects the need to re-assess the receptor status on metastasis for therapy decision [35]. However, since the metastatic site is often treated with systemic therapy and not with surgery as for the primary tumor, a biopsy is difficult to obtain.

In addition, BC is characterized by a dynamic evolution from pre-invasive stages to metastasis in which both spatial and temporal heterogeneity play a crucial role [25]. ITH is tightly related to tumor progression, resistance to therapies, and recurrence, and unveiling subclones within the tumor mass is a key challenge that could have a strong impact on clinical practice in the future [36]. Molecular investigations revealed that, besides sharing driver mutations with the primary tumor, recurrence specimens may reveal further actionable alterations [37], suggesting that a non-detectable tumor spread could be at stake at diagnosis. This point is crucial for BC management, since about 20% of patients are supposed to present tumor recurrence over the course of disease [38]. A meta-analysis showed that patients administered with adjuvant endocrine therapy for 5 years presented a recurrence risk rate ranging from 10% to 41%, further suggesting this danger [39]. In this context, a liquid biopsy approach combined with molecular analysis can be helpful to monitor BC patients for minimal residual disease (MRD) and to determine the risk of relapse [40,41], aiming at solving a challenging clinical unmet need.

## 3. CTCs and BC

CTCs constitute a rare population of cells with a crucial role in the metastatic process. Indeed, after their detachment from the primary epithelial tumor, CTCs pass through vessel endothelium to enter body circulation. The entire metastatic process remains poorly efficient, since in the bloodstream CTCs present a short half-time (approximatively between one to three hours), with only 0.1% of cells surviving after 24 hours and 0.01% having a real metastatic potential [42,43]. Indeed, in the bloodstream, CTCs are susceptible to mechanical and environmental processes, such as oxidative stress, immune system, lack of growth factors, *anoikis*, and trauma due to the blood flow stress [44]. However, the interaction of CTCs with platelets confers improved metastatic potential and protection [45,46]. 

One of the key benefits of CTC investigation concerns the possibility to individuate these cells in the bloodstream using minimally invasive techniques (i.e., blood drawn) compared to standard methods including tissue biopsy. Contextually, their investigation is useful when the biopsy of the tumor is clinically impossible or difficult to perform, for example in case of high risk of bleeding, nerve injury, or disease spreading [47]. Another important aspect concerns the opportunity to make serial withdrawals, allowing for the monitoring of the disease during treatment. Changes in CTC count proved to be clinically relevant [48,49,50]. In addition, longitudinal evaluation of CTC clusters proved to further improve prognostication as well as therapy monitoring in mBC patients starting first-line chemotherapy [51].

Up to now, the only Food and Drug Administration (FDA)-approved assay for prognosis assessment for advanced metastatic colorectal, breast, and prostate cancer patients involving CTCs is based on their raw enumeration using the CellSearch platform. In advanced BC, the presence of a number equal to or greater than 5 CTCs/7.5 mL blood is associated with unfavorable prognosis, whereas the cutoff for CTC positivity in early-stage BC is 1 CTC/7.5 mL blood, highlighting the paucity of these cell populations [14]. Even though this approach is still not comprehensive, investigation in the field of BC has demonstrated that CTC enumeration has variable prognostic value in both mBC and nonmetastatic BC (nmBC), and longitudinal investigation has further deepened their impact [50,51,52] (Table 1).

In mBC, changes in CTC levels were reported to be significantly associated with response to therapy in 58 patients treated with chemotherapy as measured by radiographic Response Evaluation Criteria In Solid Tumors (RECIST) criteria [53]. Moreover, Paoletti and colleagues recently demonstrated that CTC enumeration could be helpful to discriminate patients diagnosed with ER-positive mBC that could forgo endocrine therapy [54]. As observed in patients diagnosed with ER-positive advanced BC and treated with Palbociclib in the cTREND study, CTC enumeration during treatment can be helpful to identify early-resistance to treatment. In addition, CTC count at progression can serve as a prognostic factor to predict post-palbociclib outcome but CTC enumeration at baseline did not show any significant prognostic value [48]. However, the raw CTC enumeration does not take into account the status of these cells. Indeed, apoptotic CTCs, supposed to have an inferior metastatic potential, have been reported to be present in both mBC and nmBC patients, although more frequently in early stages [55]. Hence, evaluation of the viability of CTC could significantly improve their clinical value as proposed by Deutsch who recently found a decrease of 50% of apoptotic CTCs (M30-positive) after the first treatment cycle could detect patients with a higher risk of progression [56]. 

Concerning the non-metastatic setting, Bidard et al. found that, besides being a strong prognostic factor when detected before NAC start, detection of at least 1 CTC before NAC could accurately predict overall survival (OS) in operable or locally advanced BC patients [57]. The prognostic relevance of CTCs before and after NAC was also demonstrated in 2026 early-stage BC patients by the SUCCESS trial [52]. However, these results were not confirmed using other enumeration methods system [58], highlighting CellSearch limitations.

**Table 1:** The role of longitudinal CTC enumeration in response to therapy and prognosis.

Setting	Study	Number of Patients	Enumeration Method	Findings	Reference
**Metastatic breast cancer (mBC)**	Hartokpf	58	CellSearch	Changes in CTC levels are associated with response to chemotherapy in mBC.	[53]
	Paoletti	121	CellSearch	CTC enumeration is prognostic at baseline and follow-up in ER-positive/HER2-negative mBC patients. CTC enumeration at first follow-up identified a group of patients that could forgo endocrine therapy.	[54]
	Galardi	46	CellSearch	CTC count is a promising tool in monitoring response to palbociclib in HR-positive/HER2-negative aBC.	[48]
	Deutsch	108	CellSearch	Reduction of 50% apoptotic CTCs after one cycle of systemic therapy is a cut-off to differentiate between therapy response and disease progression; reduction <10% of apoptotic CTCs is specific for early disease progression in mBC.	[56]
**Non-metastatic breast cancer (nmBC)**	Bidard	115	CellSearch	CTC detection pre-NAC is a prognostic factor in nmBC; detection of equal or greater than 1 CTC/7.5 mL after NAC predicts OS.	[57]
	Rack	2026	CellSearch	CTC detection both before the start of systemic adjuvant treatment and after completion of chemotherapy was associated with deteriorated survival in early-stage BC.	[52]
	O’Toole	26	Screencell	Enumeration of CTC counts prior to treatment or prior to surgery is not associated with pathological response to NAC.	[58]

CTC: circulating tumor cells; ER: estrogen receptor; HR: hormone receptor; HER2: Human Epidermal Growth Factor Receptor 2; BC: breast cancer; mBC: metastatic BC; aBC; advanced BC; nmBC: non-metastatic BC; NAC: neoadjuvant chemotherapy; OS: overall survival.

Indeed, the CellSearch screening method has several drawbacks. Firstly, the number of CTCs in the bloodstream of BC patients, either early-stage and advanced, is extremely scarce as they are present in terms of few cells for milliliters of blood, and applications in clinical practice are still limited. In addition, the CellSearch system is an epithelial cell adhesion molecule (EpCAM)-based method that allows exclusively for count and isolation of epithelial CTCs, thus unable to detect mesenchymal CTCs from BC patients. This issue was raised by Satelli and colleagues [59]. In this study, cell-surface vimentin (CSV) was used as a marker for the detection of mesenchymal CTCs and combination with CellSearch further improved clinical assessment of therapeutic response. Another critical issue when using CellSearch concerns the possibility to detect circulating epithelial cells from nonmalignant colonic epithelium, as observed in patients with benign inflammatory colon diseases. Considering that benign diseases affecting the bowel are not uncommon even in oncologic patients, molecular characterization of CTCs could be helpful to detect false-positive cases [60]. Moreover, CTC enumeration provides information about the MRD without any hint on treatment choice. Recently, Sundaresan and colleagues observed that the occurrence of ESR1 mutations in CTCs from patients diagnosed with metastatic ER-positive BC is predictive of hormonal therapy response [61] supporting the role of CTCs monitoring in guiding the selection for precision medicine. Hence, since CTCs are a heterogeneous population, their in-depth molecular characterization could identify their role in the clinical setting.

### 3.1. CTCs Heterogeneity and Clinical Impact in BC

High degrees of heterogeneity at the intra- and inter-patient level have been observed in CTC from BC patients compared to normal cancer cells [62]. This heterogeneity can also lead to benefits for tumor development and impact prognosis [63]. In recent years, an increasing number of studies have attempted to achieve a clinical benefit by introducing CTC molecular characterization [64]. However, due to the technical, economic, and biological issues (i.e., intrinsic heterogeneity of CTCs), this effort remains a challenging attempt. Nevertheless, the advance in single-cell phenotypic and molecular analyses has shed some light and proved to be helpful in identifying different subpopulations [17,65]. Heterogeneity on CTCs involves different aspects, especially marker expression and molecular characteristics.

#### 3.1.1. Marker Heterogeneity

One of the well-known hallmarks of CTCs from BC patients consists of the primary and dynamic heterogeneity of expressed markers. Due to the EMT-related changes that interest CTCs and primary tumor composition, it is common to observe the emergence of many intermediate phenotypes between the epithelial and mesenchymal status [66,67,68]. As a consequence, heterogeneous CTCs can express epithelial (EpCAM, E-cadherin, cytokeratins (CKs), etc.) and mesenchymal (N-cadherin, vimentin, Zeb1, etc.) markers in a dynamic and variable manner [69], displaying different invasive and metastatic potentials. For instance, CTCs expressing epithelial marker EpCAM with a restricted mesenchymal transition were shown to have the strongest capacity to form lung metastasis compared to those expressing mesenchymal marker vimentin [70]. Multiple factors are known to influence CTC phenotypic heterogeneity, among which primary tumor subtype and treatment. For instance, in a study by Min Yu and colleagues, lobular ER and PgR positive BC patients were shown to display mainly epithelial CTCs (E-CTCs), whereas mesenchymal CTCs (M-CTCs) were more frequent in TNBC and HER2-enriched subtypes. Moreover, an increasing number of M-CTCs after treatment was associated with resistance to therapy and disease progression, as well as the occurrence of multicellular CTC clusters, as observed in one patient with lobular ER and PgR positiveBC [68]. Papadaki and colleagues observed that CTCs co-expressing stem (Aldehyde dehydrogenase 1, ALDH1) and partial-EMT related markers were enriched after first-line chemotherapy in mBC, and associated with a lack of response. In addition, detection of this CTC subset before treatment and in the HER2-negative cohort was associated with reduced progression-free survival and OS, respectively [71]. Vishnoi and collaborators observed through in vitro 3D CTC culturing CTC subsets with stem phenotype (EpCAM−/CD44+/CD24−) and combinatorial expression of uPAR and integrin β1 had different adhesive and proliferative abilities. Interestingly, gene expression analyses showed that uPAR+/integrin β1+ subset had enhanced expression of genes associated with proliferation, DNA damage repair, and brain metastasis onset [72]. More recently, D’Oronzo demonstrated that CTC from BCs patients showed four different EMT-related phenotypes (M+/E−, M+/E+, M−/E+, M−/E−), with M-CTCs the most recurrent in both naïve and pre-treated patients. Molecular analysis revealed that, besides the presence of commonly shared genetic alterations in the subset of each patient, the majority of pathogenic and non-pathogenic variants recurred in M+/E− population. Hence, the tumor clonal origin is maintained but genomic instability in M-CTCs seems to be associated with their ability to survive in the bloodstream [67]. 

#### 3.1.2. Molecular Heterogeneity

The single-cell approach enabled the stratification of CTCs in multiple populations with different competence in tumor progression and metastasis [17]. In addition to the phenotype heterogeneity, molecular heterogeneity contributes to making the picture even more complex [73]. 

For instance, Brechbul and colleagues, through a transcriptomic analysis conducted on single CTCs from mBC patients, observed the presence of different populations: one was enriched for transcripts indicative of estrogen responsiveness, whereas the other expressed increased markers associated with EMT. Strikingly, these populations had the potential to interact in different manners with peripheral blood mononuclear cells (PBMCs), and CTCs with EMT-markers were predicted for enhanced immune evasion [74]. By single-cell analysis, Kanwar et al. observed the existence of two mutually exclusive CNA signatures identifying two CTC subpopulations with different functional and metastatic abilities in BC patients. While one cluster identified chemo-resistant quiescent CTCs, characterized by fewer aberrations, involving genes such as *AKT2* and *PTEN*, the other reflected a subpopulation with extensive aberrations involving chromosome 19 and genes such as *ANGPTL4*, *BSG*, *MIR-23*, and *MIR373,* with marked metastatic ability. In addition, they identified a signature composed of 90 minimal common regions (MCRs) of copy number gain predominantly found across chromosome 19 that could regulate CTC-activity. In-depth analysis on 787 primary BC specimens revealed that these alterations were present at low frequencies (3–4%), suggesting the presence of a minor but more dangerous clone in tumor cells released in the bloodstream [75], with possible future application in dissemination risk assessment for breast and other solid tumors patients. However, the lack of data from molecular analysis still hinders the reaching of complementary information about tumor biology and heterogeneity that could be helpful in the clinical management of BC patients. 

In this context, our group, by exploiting single-cell whole-genome analysis, found that CTCs from early-stage BC patients six months after tumor resection shared CNAs with the primary tumor, suggesting the presence of regions involved in persistence mechanisms and with potential clinical impact in the future [76]. In particular, the longitudinal analysis revealed the gain of the chromosomal region hosting the telomerase reverse transcriptase encoding gene (TERT) in CTCs and primary tumor specimen of one TNBC patient. However, patients did not experience tumor progression, suggesting that CTCs did not have short-term metastatic potential, as a confirmation that CTC behavior could be different. Moreover, the first investigation of CTCs of a rare and aggressive subtype, metaplastic spindle-shaped BC, revealed that besides the high CTC count (>200 CTCs in 7.5 mL blood), great degrees of heterogeneity were present at both molecular and phenotypic levels, inferring that molecular characterization of CTCs could have a role in understanding the mechanisms underlying the metastatic process [77]. 

Single-cell CNA analysis revealed that single CTCs from patients BC with brain metastasis genomically resembled primary tumor, and alterations in pathways known to be implied in brain metastasis formation (i.e., notch and PI3K pathways) were also present. Riebensahm et al. also showed that single CTCs were highly clonal among cells from the same patient, hypothesizing that brain metastasis competence could be due to clonal selection [78]. Moreover, CNAs and mutational analysis in mBC endocrine-resistant patients by Paoletti and colleagues demonstrated high concordance levels (>85%) in at least one or more recurrent somatic mutations and CNAs between CTCs and matched metastasis biopsies. Simultaneously, patients in which discordances were observed displayed actionable alterations exclusively present either in CTCs or in metastasis, supporting the potential gaining of complementary tumor information through CTC analysis. Heterogeneity of estrogen receptor 1 encoding gene (*ESR1*) mutations were evident in single CTCs, demonstrating the presence of subclones involved in resistance to endocrine therapies in multiple ways [79].

## 4. Conclusions

Up to now, BC represents a critical health issue worldwide, also due to its high degrees of heterogeneity, both at the inter- and intra-patient level. Consequences of ITH involve the presence of subpopulations within the same tumor displaying heterogeneous angiogenic, invasive, and metastatic traits, hence modulating, and potentially extending in time, the risk of disease recurrence [80]. Indeed, as reported by a meta-analysis published by the Early Breast Cancer Trialists’ Collaborative Group (EBCTCG), relapse may occur even after decades, also affecting patients with small tumors (T1) and with negative axillary lymph nodes (N0) [39,81]. Moreover, it is well known that ITH is a major determinants in therapy resistance and treatment failure in all cancer types including BC [82,83]. However, since the routinely applied approach for BC assessment (i.e., needle biopsy) systematically fails to unmask ITH, other tools have been proposed and are worthy of further application. In particular, the analysis of CTCs proved to be a promising tool in this context, and many studies suggested including their investigation in the clinical practice [84,85,86]. In agreement, this review, reporting the latest findings concerning the phenotypic and molecular characterization of CTCs in overcoming the ITH issue in BC, helps to infer that their assessment should be integrated into the clinical practice.

Considering all the highlighted aspects, it is clear that some deficiencies in present CTC-based assays require attention. In addition to phenotypic diversity, molecular heterogeneity could provide information for therapy monitoring and follow-up to improve personalized medicine in BC. Since NGS applications on CTCs imply high costs, including these analyses in clinical practice is still challenging. Different studies in the literature have demonstrated that whole-genome sequencing and RNA-sequencing could identify signatures associated with metastasis and provide information concerning CTC organotropism [72,75,78,87]. These data have the potential to drive the generation of targeted analyses on patients’ CTCs, making their investigation more feasible and economically applicable to the clinical setting. However, before moving from bed to bench side, future prospective validations are required.

Together with detection, molecular characterization of CTCs could close the gap concerning the follow-up in nmBC patients. Indeed, the current guidelines include yearly mammography and instrumental tests in case of symptoms [88], taking the risk of ignoring asymptomatic metastatic occurrence [89]. 
